# Dual wave of neutrophil recruitment determines the outcome of *C. albicans* infection

**DOI:** 10.3389/fcimb.2023.1239593

**Published:** 2023-07-10

**Authors:** Weiwei Zhu, Huifang Zhang, Qiming Dong, Hongyong Song, Lin Zhao

**Affiliations:** ^1^ Department of Cardiovascular Medicine, Fifth Affiliated Hospital of Sun Yat-sen University, Zhuhai, Guangdong, China; ^2^ Department of Cardiology, Beijing Anzhen Hospital, Capital Medical University, Beijing, China; ^3^ Department of Internal Medicine, Greater Baltimore Medical Center, Towson, MD, United States; ^4^ Department of Cardiovascular Medicine, Central Hospital Affiliated to Shandong First Medical University, Jinan, Shandong, China

**Keywords:** *Candida albicans*, neutrophils, LFA-1, Mac-1, ICAM-1

## Abstract

*Candida albicans* is a ubiquitous fungus that can cause superficial and systemic infections in humans. Neutrophils play a crucial role in controlling *C. albicans* infections. When *C. albicans* enters the bloodstream, it tends to get trapped in capillary vessels. However, the behavior of neutrophils in combating capillary-residing fungi has not been fully characterized. In this study, we used transgenic mice and whole mount imaging to investigate the growth of *C. albicans* and its interaction with innate immune cells in different organs. We observed that *C. albicans* rapidly grows hyphae within hours of infection. Following intravenous infection, we observed two waves of neutrophil recruitment, both of which significantly contributed to the elimination of the fungi. The first wave of neutrophils was induced by complement activation and could be prevented by C5aR blockade. Interestingly, we discovered that the fungicidal effect in the lungs was independent of adhesion molecules such as Mac-1, LFA-1, and ICAM-1. However, these molecules played a more significant role in the optimal killing of *C. albicans* in the kidney. Importantly, the initial difference in killing efficiency resulted in significantly reduced survival in knockout mice lacking these adhesion molecules. We identified a second wave of neutrophil recruitment associated with hyphal growth and tissue damage, which was independent of the aforementioned adhesion molecules. Overall, this study elucidates the dual wave of neutrophil recruitment during *C. albicans* infection and highlights the importance of early fungal clearance for favorable disease outcomes.

## Introduction

The fungal pathogen *Candida albicans* (*C. albicans*) is recognized as a major cause of candidiasis (Netea et al., 2015). The World Health Organization has recently classified *C. albicans* as a critical priority fungal pathogen, highlighting its significance in global public health (Chowdhary et al., 2017). Another critically listed *Candida* species is the emerging *Candida auris*, however, *C. albicans* remains the most prevalent fungal pathogen in clinical settings (Pappas et al., 2018).

*C. albicans* is a fungus found ubiquitously in the environment and is commonly detected on human mucosal surfaces, making it a part of the normal human microflora (Kullberg and Arendrup, 2015; Swidergall and LeibundGut-Landmann, 2022; Anderson et al., 2023; Du Toit, 2023). Various body locations including the skin (Kashem and Kaplan, 2016), reproductive tract (Wira et al., 2011; Salinas-Munoz et al., 2018), gastrointestinal system (Kumamoto et al., 2020), and respiratory system (Neville et al., 2015) can harbor *C. albicans*. In immunocompetent individuals, the commensal relationship between *C. albicans* and the host generally does not lead to health issues (Pappas et al., 2018). However, disruption of the normal host-pathogen balance, such as in immunosuppressed individuals or patients with catheters, can result in *C. albicans* infections. *C. albicans* is capable of causing both mucosal and systemic infections (Kullberg and Arendrup, 2015; Netea et al., 2015). The sources of *C. albicans* leading to candidemia have been debated, and more recent studies suggest that the gut serves as the origin of disseminated fungi (Nucci and Anaissie, 2001). Dissemination of *C. albicans* into the bloodstream results in invasive candidiasis, the most severe form of infection. Various organs can be affected during invasive candidiasis, including the kidney (Netea et al., 2015), the heart (Mamtani et al., 2020) the brain (Forster et al., 2022), etc. *C. albicans* infection of the heart leads to endocarditis (Mamtani et al., 2020), which has a six-month mortality rate of approximately 25% (Park et al., 2016).

Several virulence factors contribute significantly to the pathogenesis of *C. albicans*, with the morphological switch from yeast to filamentous form being a key feature (Gow et al., 2011). While the fungus typically grows in the yeast form in nutrition-rich environments, it can transform into the hyphal form under specific nutritional conditions. The morphological change, including the growth of hyphae or pseudohyphae, is associated with differentially expressed surface markers that affect host immune cell recognition and responses (Wheeler et al., 2008). Additionally, *C. albicans* produces various secreted factors, such as aspartyl proteases, phospholipases (Felk et al., 2002) and the cytolytic toxin candidalysin (Moyes et al., 2016) which aid in invasive infection. Effective adherence to endothelial and epithelial cells, as well as biofilm production, also significantly contribute to the pathogenesis of *C. albicans*, enhancing its antifungal resistance and ability to cause recurrent infections (Desai et al., 2014).

Innate immunity, particularly neutrophils, plays a critical role in defense against *C. albicans* infections. Neutropenic patients with reduced neutrophil counts or those with defective neutrophil function, such as those with chronic granulomatous diseases (CGD, ROS production deficiency due mutations in NAPDH oxidase subunit), are more susceptible to *C. albicans* infections (Henriet et al., 2013). Neutrophils exert their antifungal effects through various mechanisms, including phagocytosis, degranulation, ROS production, and the formation of neutrophil extracellular traps (NETs) (Wu et al., 2019; He et al., 2022). They can sense the size of the *C. albicans* and selectively release NETs in response to large pathogens, such as the *C. albicans* hyphae form (Branzk et al., 2014). *C. albicans* is capable of inducing NETosis through NADPH oxidase-dependent or -independent pathways (Wu et al., 2019). NETs have been shown to effectively kill both the yeast form and hyphal form of *C. albicans*. Many receptors have been identified to recognize *C. albicans*. Various receptors have been identified to recognize *C. albicans* (Netea et al., 2006; Bojang et al., 2021), with Dectin-1 and DC-SIGN (Cambi et al., 2003) being the most well-characterized receptors expressed on neutrophils and dendritic cells, respectively. The receptors Dectin-1 (Philip R Taylor et al., 2007) and Mac-1 (CD11b/CD18) on neutrophils play a crucial role as they act as receptors for *C. albicans* and are required for optimal neutrophil killing of the fungus (Li et al., 2011). Activation of Dectin-1 leads to inside-out activation of integrins Mac-1 through Vav protein signaling (Dupuy and Caron, 2008). In addition, Dectin-1 mediated phagocytosis acts as a sensor of microbe size and prevents NET release by inhibiting the translocation of neutrophil elastase to the nuclei (Branzk et al., 2014).

The host has developed various pathways to mobilize and maintain neutrophils, which are essential for host defense against invading pathogens. Circulating neutrophil counts can be positively regulated by CXCL1-CXCR2 signaling and negatively regulated by the CXCR4-CXCL12 signaling. Additionally, IL-17 is important for regulating neutrophil responses, and previous studies have shown both innate and adaptive sources of IL-17 in response to mucosal *C. albicans* infection (Kashem and Kaplan, 2016). In other fungal infections, PD-L1 has been found to negatively regulate antifungal immunity by inhibiting neutrophil release from the bone marrow (Yu et al., 2022). What factors regulate neutrophil recruitment during systemic *C. albicans* infection has not been completely revealed.

Under normal conditions, neutrophils are present in large numbers in the circulation. Previous studies have indicated that circulating neutrophils undergo a process of rolling, adhesion, crawling, and extravasation to perform their functions. However, these studies have primarily focused on post-capillary venules, with the cremaster muscle (Phillipson et al., 2006) or ear dermis (Ng et al., 2011; Kleinholz et al., 2021) often used as study subjects. During invasive candidiasis, *C. albicans* is typically trapped in capillaries due to its size and solid structure. Despite numerous studies conducted in the past decades on the pathogenesis of *C. albicans*, the detailed interactions between host neutrophils and the fungi have not been fully elucidated. Therefore, the objective of this study was to investigate whether and how adhesion molecules are involved in the anti-fungal effects during systemic candidiasis.

## Materials and methods

### Animals

Wild type C57BL/6 mice were obtained from Charles Rivers. Transgenic mice, including CD11b^-/-^ (Cat# 003991), CD11a^-/-^ (Cat# 005257), ICAM-1^-/-^ (Cat# 002867), and CX3CR1^gfp/gfp^ (Cat# 005582), were purchased from Jackson lab and bred in specific pathogen-free (SPF) level animal facility of our institute. The mice were housed in controlled conditions with a temperature of 22-24°C, humidity of 30-60%, and a 12-hour light/12-hour dark cycle. Mice aged 6-12 weeks (both male and female) were used for all experiments. The animal study protocol was approved by the Animal Ethics Committee of Sun Yat-Sen University under protocol number [2018]08–81. The guidelines for the Ethical Review of Laboratory Animal Welfare (GB/T35892-2018) and institutional ethical guidelines for animal experiments were followed.

### Fungal strains

The *C. albicans* (SC5314 strain) used in this study was purchased from the American Type Culture Collection (ATCC). *C. albicans* from glycerol stocks were inoculated into 15 ml YPD medium and cultured in a shaking incubator at 30°C for 12 hours until the log phase was reached. The cells were then collected by centrifugation at 500 g for 5 min and washed twice with sterile PBS. The fungal cells were enumerated using a hemocytometer.

### Animal model

Mice were infected with 10^5^ or 10^6^ freshly collected yeast form *C. albicans* via the lateral tail vein, unless otherwise stated. After infection, mice were weighed and assessed for pain on a daily basis. Mice were euthanized if they lost up to 20% of their body weight. In some experiments, *C. albicans* cells were stained with 0.05% Uvitex 2B (Polysciences) dye to label the fungal cell wall. To study the dynamics of fungi after intravenous (i.v.) injection, 10 μl of blood was collected from the tail vein and plated on YPD agar for enumeration.

### Quantification of fungal burden in organs

At designated time points after infection, both kidneys and other organs were collected in 15 ml tubes and homogenized with a tissue homogenizer (Omni International) in 2 ml of RPMI 1640 medium. The homogenate was serially diluted and plated on YPD agar. The plates were incubated at 30°C for 36 hours, and the resulting colonies were enumerated.

### Whole mount confocal imaging

For imaging the growth of *C. albicans* in different organs, mice were infected with prelabeled *C. albicans*, either with 0.05% Uvitex 2B or 10 μg/ml FITC. The labeled fungi were injected via the tail vein at a concentration of 10^6^ per mouse. At designated times, the kidneys and brain were collected and directly imaged using an inverted confocal microscope (Zeiss LSM800) with a 63x objective for the highest resolution. The images were analyzed using ZEISS ZEN 3.0 (blue edition) software. Z projection images were generated for organ imaging. In cases where confocal microscopy was not feasible for imaging large quantities, an inverted Nikon Ti2 microscope was used, and the images were analyzed using FIJI software.

### Analysis of *C. albicans* phagocytosis by immune cells

To study the phagocytosis by macrophages or neutrophils, immunofluorescence study of F4/80 (clone BM8) and Ly6G (clone 1A8) was performed. Briefly, mice were infected with 10^6^ prelabeled *C. albicans* for 30 min, organs were fixed by 4% paraformaldehyde after collection and snap frozen followed by cryosection into 7 μm thick sample. Staining was performed by first antibody (1:200 dilution from Biolegend) at 4°C for 12 hours and Alex Fluor 555 Goat-anti rat second antibody (Abcam, 1:500). Phagocytosis was defined by colocalization of immune cells with fungi.

### Cytokine measurement

Cytokines were measured using ELISA kits for the detection of specific cytokines purchased from R&D Systems (TNF-α and C5a) and eBiosciences (IL-1β), following the manufacturers’ instructions. Freshly excised organs were placed in 15 ml tubes containing 2 ml of PBS and kept on ice. Lung tissues were homogenized using a hand-held tissue homogenizer (Omni) and centrifuged at 4000 g for 5 min. The supernatants were collected for cytokine measurement.

### C5aR blockade

To block C5aR signaling, anti-C5aR antibody was purchased from Biolegend, and injected into the tail vein of mice 30 min before the infection of fungi at a dosage of 100 μg/mouse.

### Immune cell isolation and flowcytometry

Mice were euthanized by CO_2_ exposure, and the organs were harvested and placed on ice. After collecting all groups, the organs were minced into tiny pieces using fine surgical scissors and digested with 1 mg/ml Collagenase IV and 1 mg/ml DNase I at 37°C for 40 min. The digest was then forced through 70 μm cell strainers using a 5 ml syringe plunger. The filtrates were centrifuged at 400 g for 5 min to remove cell debris. After removing the supernatant, the cell pellets were resuspended in 2 ml of 30% Percoll (GE HealthCare) and carefully layered onto 80% Percoll in a 15 ml tube without disturbing the interface. The tube was then centrifuged at 1000 g for 15 min at room temperature. The cells at the interface between the 30% and 80% layers were collected and washed with flow staining buffer (1% BSA with 0.05% sodium azide). Isolated immune cells were counted using a hemocytometer, and 10^6^ cells were collected for further analysis. Prior to staining, cells were blocked with Fc receptor blocking antibody at room temperature for 30 min and stained with the appropriate antibodies. All antibodies (CD45 APC-Cy7, CD11b PE-Cy7, Ly6C PerCP, Ly6G AF647, F480 FITC, NK1.1 PE) were purchased from BioLegend. The cells were analyzed using a FACS Aria III flow cytometer. The resulting FCS file was analyzed using FlowJo V10 software.

### ROS determination

The generation of reactive oxygen species (ROS) in neutrophils was measured using Dihydrorhodamine 123 (D23806, ThermoFisher) according to the manufacturer’s instructions.

### Statistics

All data were expressed as mean ± SEM. GraphPad Prism 5 software (San Diego, CA) was used for data analysis and figure plotting. For determination of significance, unpaired two-sided Student’s *t*-test was performed for single comparisons, while one-way analysis of variance (ANOVA) followed by Bonferroni’s *post hoc* test was used for comparisons involving more than two groups. In both cases, *p* < 0.05 was considered significant.

## Results

### Yeast form of *C. albicans* get trapped in capillary

**Figure 1:**
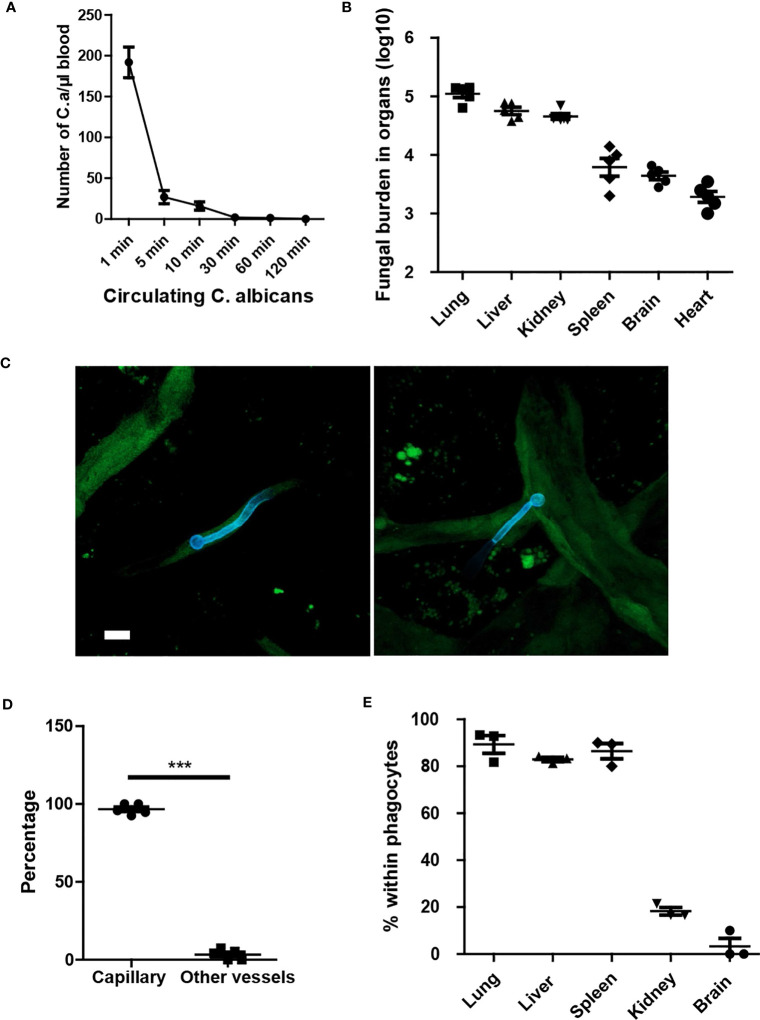
Yeast form of *C*. *albicans* are rapidly cleared from circulation and trapped in capillary. **(A)** Mice (n=5) were treated with 1x10^6^ *C*. *albicans* and the number of circulating fungi was enumerated at different time points using tail vein blood. **(B)** 10 min after infection, the distribution of fungi in different organs were evaluated. **(C)** Whole mount confocal images of *C*. *albicans* in the kidney 4 hours after infection. **(D)** The number of *C*. *albicans* with body residing in capillary vessels. capillary vessel was defined as diameter smaller than 10 μm. **(E)** The enumeration of *C*. *albicans* within phagocytes, phagocytes were stained by F4/80 and Ly6G to distinguish macrophage and neutrophils. CX3CR1^gfp/+^ mice were used to identify resident macrophage in the kidney and brain. Scale bar =10 μm. ***, *p*<0.001 by student’s t test.

To investigate the dynamics of *C. albicans* movement in the host, we injected *C. albicans* into the tail vein and monitored the clearance of the fungi within the circulation. The results indicated that *C. albicans* were rapidly removed from circulation (**Figure 1A**). Enumeration of fungal burden in different organs revealed that the lungs captured the majority of fungi, followed by the liver (**Figure 1B**). Subsequently, we imaged the fungi in the kidney and observed that *C. albicans* in the yeast form grew hyphae within the first few hours (**Figure 1C**). Notably, *C. albicans* primarily localized in the capillary vessels (**Figure 1D**). Further investigation revealed rapid phagocytosis of *C. albicans* by resident macrophages in the liver and spleen, and by neutrophils in the lungs (**Figure 1E**). Phagocytosis of *C. albicans* in the brain and kidney occurred at a relatively slower rate.

### Organ specific response to *C. albicans* immediately after infection

**Figure 2:**
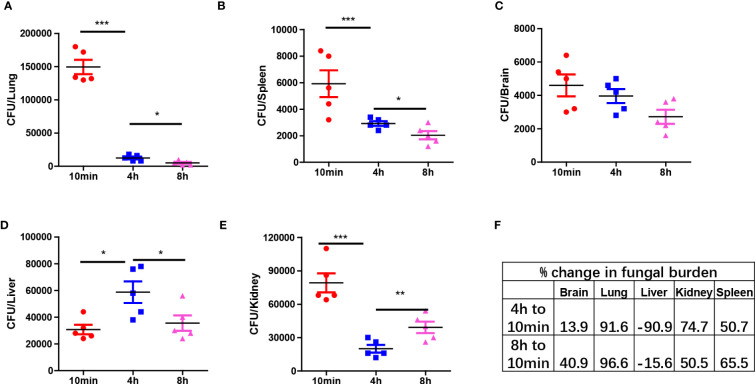
Organ specific response to *C. albicans* immediately after infection. **(A–E)** C57BL/6 mice were infected with 1x10^6^ *C. albicans*, mice were euthanized at three different time points and the fungal burden in different organs were enumerated. **(F)** The percent change of fungal burden in different organs by comparing CFU at 4 h and 8 h to 10 min. negative value means CFU increased comparing to 10 min. *p<0.05; ***p<0.001 by one-way ANOVA.

Next, we analyzed the clearance of *C. albicans* in different organs at three time points: 10 min, 4 hours, and 8 hours post-infection. The results demonstrated distinct antifungal effects in different organs, with a significant portion of yeast-form fungi being cleared from most organs within hours. Organs such as the lungs, spleen, and brain exhibited a constant reduction in fungal burden, indicating the killing of *C. albicans* in those organs (**Figures 2A–C**). However, in the liver, the fungal burden initially increased and then decreased (**Figure 2D**). In contrast, the fungal burden in the kidneys first decreased and then rebounded at 8 hours, showing an opposite trend to that in the liver (**Figure 2E**). Calculation of clearance efficiency revealed that the lungs exhibited the fastest antifungal efficiency, while the brain was comparatively less efficient (**Figure 2F**). These findings confirm the existence of organ-specific immune responses to pathogens.

### *C. albicans* i.v. infection triggers complement dependent first wave of neutrophils

**Figure 3:**
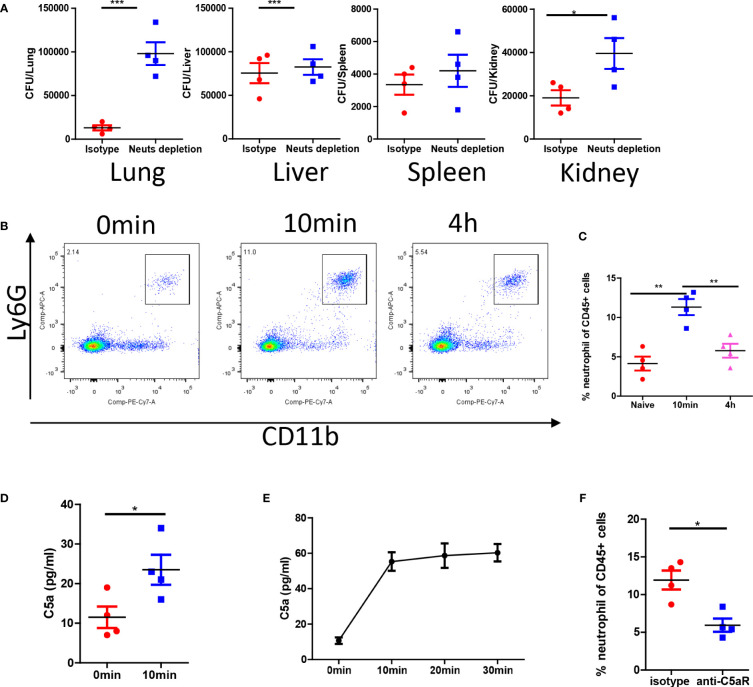
C. *albicans* i.v. infection triggers C5aR dependent neutrophils release. **(A)** C57BL/6 mice were treated with 200 μg/mice anti-Ly6G antibody or isotype control and infected with 1x10^6^ *C*. *albicans*. The fungal burden in different organs were evaluated 4 h after infection. **(B)** Blood leukocytes were studied for the percentage of neutrophils at naive, 10min and 4h post infection. **(C)** The statistics of neutrophil percentage after infection. **(D)** The release of C5a in the serum 10min after infection comparing to naive. **(E)** *In vitro* release of C5a when *C*. *albicans* were incubated in the serum (n=3). **(F)** C57BL/6 mice were treated with 100 μg/mice anti-C5aR antibody or isotype control and infected with 1x10^6^ *C*. *albicans*, the percentage of neutrophils in the circulation was studied. *, *p*<0.05; ***, *p*<0.001 by student’s *t* test or one way ANOVA **(C)**. **p<0.01 by student’s t test.

The antifungal effect observed in the pulmonary vasculature was particularly remarkable. Neutrophil depletion experiments confirmed that the antifungal effects were mediated by neutrophils in the lungs but not in the spleen and liver (**Figure 3A**). *C. albicans* infection promptly induced the release of neutrophils into circulation (**Figure 3B**). Neutrophil release peaked minutes after infection and gradually declined after several hours (**Figure 3C**). *C. albicans* rapidly activated complement and released C5a fragments both *in vivo* (**Figure 3D**) and *in vitro* (**Figure 3E**). To demonstrate the role of C5a in neutrophil release, we treated mice with a C5a receptor (C5aR) blocking antibody, which successfully inhibited neutrophil release into the circulation (**Figure 3F**). In summary, our findings suggest that *C. albicans* triggers immediate complement activation through C5aR signaling upon infection.

### Adhesion molecules is required for anti-fungal response

**Figure 4:**
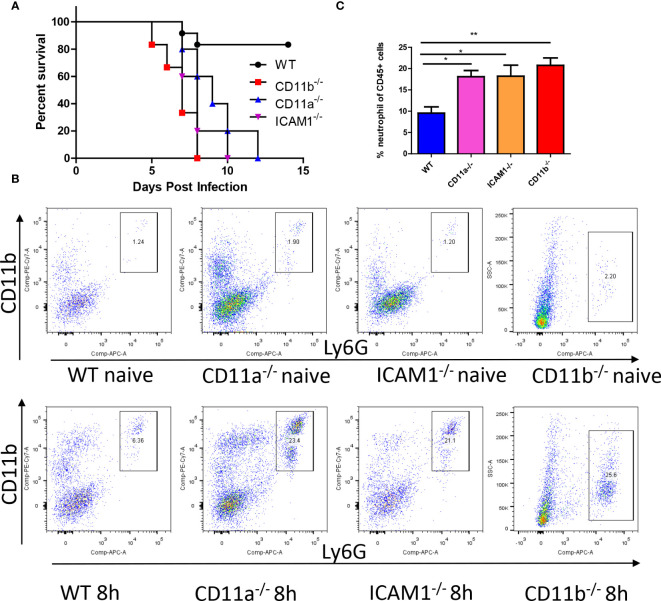
Adhesion molecules is required for anti-fungal response. **(A)** WT mice and mice deficient for CD11b, CD11a and ICAM-1 were infected with 10^5^ *C. albicans*, their survival curve was evaluated. **(B)** Mice with or without infection with 10^6^ C*. albicans* for 8h were euthanized, the recruitment of neutrophils (CD45^+^CD11b^+^Ly6G^+^) in the kidney was analyzed by flowcytometry. Note the y axis for CD11b mice is side scatter instead of CD11b due to knockout of this gene. **(C)** The statistics of neutrophil percentage out of total leukocytes (CD45^+^ cells, n=4). *, *p*<0.05; **, *p*<0.01 by student’s *t* test.

Considering the importance of adhesion molecules in neutrophil recruitment and function, we further investigated their roles in the fungicidal effects. We infected wild-type (WT) mice along with CD11b^-/-^, CD11a^-/-^, and ICAM1^-/-^ mice with *C. albicans* and compared their survival rates. The results demonstrated reduced survival in all knockout (KO) mice after infection (**Figure 4A**). Host mortality was attributed to uncontrolled growth of *C. albicans* in the kidneys (**Figure S1**). We next studied the recruitment of neutrophils and their anti-fungal effects. By analyzing neutrophils after infection, we were surprised to find neutrophils were even increased in these KO mice (**Figures 4B, C**).

Since CD11b/CD18 forms complement receptor 3, which can bind to C3b/iC3b on pathogen surfaces, we studied whether CD11b/CD18 is required for liver capture of *C. albicans*. We injected WT and CD11b^-/-^ mice with *C. albicans* and monitored the distribution of *C. albicans* in the host. Interestingly, we found that CD11b^-/-^ mice displayed an increased fungal burden in the liver (**Figure S2**), suggesting that CD11b is not required for liver capture of *C. albicans*. The increase in liver colony-forming units (CFU) is likely due to reduced killing in other organs.

### *C. albicans* hyphae growth induces second wave of neutrophils

**Figure 5:**
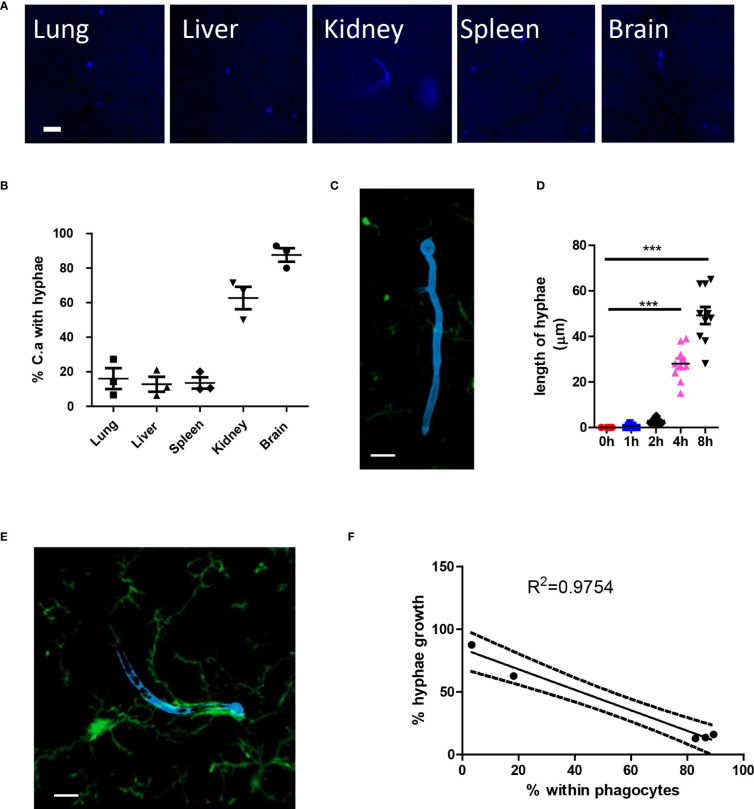
*C. albicans* hyphae growth induces second wave of neutrophils. **(A)** WT mice were infected with 10^6^ *C. albicans* labeled with Uvitex 2B, the growth of hyphae in different organs was studied by fluorescence microscope. **(B)** The percentage of *C*. *albicans* with hyphae growing at 4 h post infection. **(C)** Whole mount confocal images showing the penetration of tissue by candida hyphae. **(D)** The length of longest hyphae grows with time in kidney. **(E)** Whole mount confocal images showing the growth of candida hyphae and association with brain resident macrophage. **(F)** The regression curve of percent within phagocytes versus percent hyphae growth, five dots represent different organs, the lung, brain, spleen, kidney, and liver. Scale bar=10 μm. ***, *p*<0.001 by student’s *t* test.

After infection, *C. albicans* grows hyphae in organs; however, the growth of hyphae can be inhibited in different organs (**Figures 5A, B**). More *C. albicans* grows hyphae in the kidney and the brain, while most fungi in the lung, liver, and spleen remain inhibited. High-resolution confocal images revealed that the growth of hyphae penetrates deep into the organ (**Figure 5C**), and the hyphae can reach lengths of up to 60 μm, which is more than ten times the normal diameter of yeast form *C. albicans* (**Figure 5D**). Previous reports have shown that kidney resident macrophages are able to respond to invading *C. albicans* in the kidney within 2 hours. Here, we demonstrated that brain resident macrophages can also respond to *C. albicans* (**Figure 5E**). However, this association does not seem to affect hyphal growth. Moreover, we showed that the percentage of phagocytosis is inversely correlated with the percentage of hyphae growth in the five analyzed organs (**Figure 5F**).

### Second wave of neutrophil is associated with fungal burden

**Figure 6:**
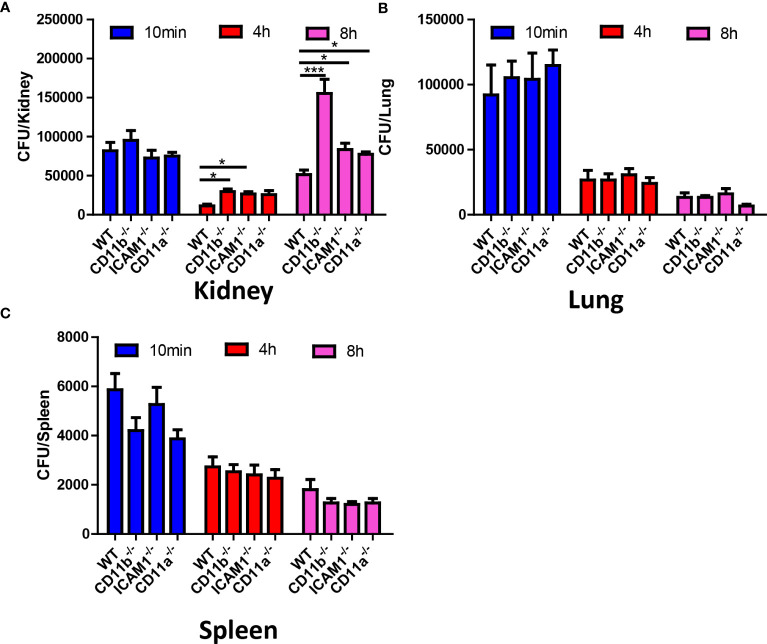
Fungal burden of WT, CD11b, CD11a and ICAM-1 KO mice. Mice (n=4) were infected with 10^6^ *C. albicans*, and euthanized at 10 min, 4 h and 8 h, the fungal burden in the kidney, the lung and the spleen were evaluated. **(A)** Kidney, **(B)** Lung, **(C)** Spleen. *, *p*<0.05; ***, *p*<0.001 by student’s *t* test.

To address the role of the aforementioned adhesion molecules in neutrophil recruitment and their antifungal capability, we compared the fungal burden in CD11b^-/-^, CD11a^-/-^, and ICAM1^-/-^ mice in different organs, with a focus on the kidney. The results suggested that all KO mice have an increased *C. albicans* burden in the kidney at 8 hours (**Figure 6A**). In fact, the difference in kidney fungal burden is already noticeable as early as 4 hours post-infection (**Figure 6A**). Surprisingly, we found that all KO mice killed an equivalent number of fungi in the lung and spleen (**Figures 6B, C**), including CD11b^-/-^ mice. CD11b-deficient neutrophils have been shown to have reduced phagocytosis and reactive oxygen species (ROS) production (Li et al., 2011). However, the neutrophils from these KO mice showed equivalent production of ROS after stimulation with PMA, suggesting they have the potential for oxidative burst (**Figure S3**). Collectively, these results emphasize the importance of early killing of *C. albicans*. The slight difference in killing efficiency in the kidney can translate into significantly reduced survival later on.

### CD11b, CD11a and ICAM-1 is required for optimal recruitment of first wave of neutrophils in kidney

**Figure 7:**
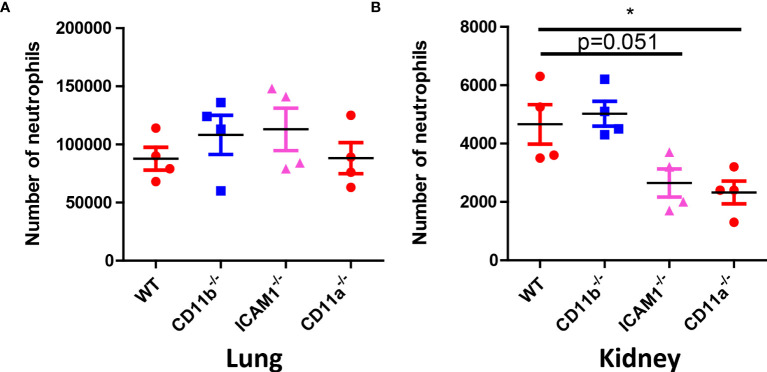
Neutrophil counts in WT, CD11b, CD11a and ICAM-1 KO mice. **(A)** Mice (n=4) were infected with 10^6^ *C. albicans* for 30 min, the lung and the kidney were collected for neutrophil analysis by flow cytometry. **(A)** Lung, **(B)** Kidney. *, *p*<0.05 by student’s *t* test.

The above results suggested early removal of *C. albicans* in the kidney in crucial for future survival. Neutrophil requires adhesion molecules for optimal recruitment. Although neutrophil recruitment at later time point does not depend on CD11b, CD11a and ICAM-1, we hypothesized that these molecules are important for the early recruitment of neutrophils to the kidney and optimal killing. To prove this hypothesis, we studied the recruitment of neutrophils 30 min after infection in the lung and kidney, the results showed that while neutrophil recruitment in the lung does not require the adhesion molecules (**Figure 7A**), their recruitment to the kidney at the early stage need adhesion molecules CD11a and ICAM-1 (**Figure 7B**).

## Discussion

The most devastating form of *C. albicans* infection is systemic candidiasis which happens when the commensal fungi are released into the blood stream and invade internal organs (Romo and Kumamoto, 2020). The size of yeast form *C. albicans* (around 4-5 μm) is close to the inner diameter of most capillary vessels. In contrast to blood cells which can change their shape and squeeze through narrow vessels. *C. albicans* cannot change its shape and are rapidly captured in the capillary vessels of different organs. Entering the circulation is the first step for the development of invasive candidiasis, thus the i.v. injection route used in the study closely mimics the clinical situation of systemic candidiasis, where *C. albicans* is released into the bloodstream and invades internal organs.

Innate immunity plays a key role in the defense against *C. albicans*. Both neutrophils and macrophages contribute to the antifungal effect. The balance between innate immunity, particularly neutrophils and mononuclear phagocytes, and *C. albicans* determines the outcome of systemic candidiasis. Neutrophil is the most well-characterized host defense mechanism against *C. albicans* infection (Netea et al., 2015; Wu et al., 2019; He et al., 2022). Consistent with previous knowledge, our study also highlights the significance of the early-stage antifungal effect mediated by neutrophils, emphasizing its importance for predicting the future outcomes of infection.

Microglia has been reported to mediate protective neutrophil recruitment to the fungus-infected brain via IL-1β and CXCL1 (Drummond et al., 2019). Previous studies primarily used immunohistochemistry to study *C. albicans* within the host. In this study, fluorescently labeled fungi and whole-mount confocal imaging were utilized to unveil the growth of *C. albicans* in the host and its pathogenicity. We provided high-resolution images showing the interaction of *C. albicans* and brain microglia at the early stage of infection in the brain.

Previous reports suggested that kidney tissue resident mononuclear phagocytes interact with *C. albicans* early after infection, with approximately 90% association observed within two hours (Lionakis et al., 2013). They further showed these renal tissue resident macrophages rely on CX3CR1 expression for survival (Lionakis et al., 2013). Although this macrophage association is considered rapid, it is slower than those happened in the liver and the spleen, where a large number of professional phagocytic macrophages are present. Furthermore, while resident macrophages in the kidney can inhibit and kill *C. albicans*, their efficiency appears to be lower than that of liver and spleen macrophages, which exhibit stronger antifungal capabilities. Our results suggest that phagocytosis in the early stages of infection is more effective in preventing *C. albicans* growth.

Our study demonstrates that phagocytosis significantly inhibits hyphal growth. In the lung, *C. albicans* induces significant neutrophil accumulation in pulmonary vasculature, and the fungi are rapidly phagocytized by neutrophil. In the liver and spleen, the presence of the mononuclear phagocyte system (historically known as the reticuloendothelial system or RES) allows efficient engulfment of *C. albicans*. Phagocytosis occurs rapidly within one hour post-infection, with over 90% of the fungi being phagocytized in the spleen, liver, and lung. Although phagocytosis also occurs in the kidney and brain, the rate of phagocytosis in these organs is slower compared to the aforementioned organs. Consequently, the kidney and brain exhibit the highest percentage of hyphal growth. It is important to note that although a large portion of *C. albicans* associates with resident macrophages in the kidney and brain, this association does not seem to inhibit hyphal growth effectively. Thus, there seems a huge difference in terms of killing efficiency comparing macrophage in the liver with macrophages in the kidney and the brain. Future studies are need to identify the factors contributing to the different killing efficiency.

It is worth to note that the current work emphasized the dramatic difference in immune responses of different organs, which lead to different anti-fungal behavior. We showed that due to the existence of marginal neutrophil population in the lung and the rapid activation of complement system, *C. albicans* in the lung triggered almost immediate neutrophil retention in the lung that is not dependent on adhesion molecules Mac-1, LFA-1 and ICAM-1. Previous studies have shown that lung marginated neutrophil population and splenic resident neutrophils are pioneers in host defense against systemic bacterial infection (Devi et al., 2013; Juzenaite et al., 2021). Interestingly, while CD11b has been reported to be essential for optimal killing of *C. albicans* (Li et al., 2011), our study shows that mice lacking Mac-1 can still effectively eliminate fungi in the lung. This implied that the unique vascular structure and recruitment strategy of the lung contribute to this differential response.

Although LFA-1 and Mac-1 are well-studied adhesion molecules that mediate distinct cellular processes during leukocyte trafficking, previously reported have shown that they play distinctive roles in leukocyte adhesion and crawling (Hyun et al., 2019). For example, Phillipson et al. demonstrated that neutrophils trafficking involves LFA-1 dependent adhesion followed by Mac-1 dependent crawling (Phillipson et al., 2006). Other studies proposed that LFA-1 is required for firm neutrophils attachment during increased shear stress (Ding et al., 1999). It is important to note that the function of adhesion molecules may vary depending on the cell type. In monocytes and lymphocytes, LFA-1 has been shown to be involved in crawling and transendothelial migration (Auffray et al., 2007; Shulman et al., 2009). Our result suggested that CD11a (LFA-1) and ICAM-1 is required for early recruitment of neutrophils to the kidney, but not CD11b. However, we could not exclude the possibility that recruited CD11b^-/-^ neutrophils is deficient in crawling or other behavior. Future studies focusing on early-stage intravital imaging can provide more insights into the exact functions of these molecules during the anti-fungal response.

Candidalysin is known to play a role in neutrophil recruitment during systemic *C. albicans* infection (Swidergall et al., 2019). The hyphae form of *C. albicans* can crucially lead to NLRP3 inflammasome activation via candidalysin (Rogiers et al., 2019), leading to cytolysis in mononuclear phagocytes (Kasper et al., 2018). Whether there is any difference in the dual wave neutrophil recruitment in candidalysin and hyphae deficient *C. albicans* still requires further study.

In conclusion, our study showed systemic *C. albicans* infection triggers dual wave of neutrophils that is associated with organ specific anti-fungal responses. Although fungal killing happens in all organs, *C. albicans* cannot be eradicated thoroughly. Instead, the fungi changed into a persistent form and reside in the tissue for prolonged time. Our study implied that, the initial early-stage the killing before hyphae growth is important for the disease outcomes.

## Data availability statement

The original contributions presented in the study are included in the article/**Supplementary Material**. Further inquiries can be directed to the corresponding authors.

## Ethics statement

The animal study was reviewed and approved by Sun Yat-Sen University.

## Author contributions

WZ, HS, and LZ contributed to the conception and design of the research, WZ and HZ completed the experimental work, prepared for the initial manuscript draft. WZ, QD and HZ performed experiments and analyzed data. WZ, HZ, LZ, and HS contributed to the conception and design of the research and manuscript development, editing and revisions. All authors contributed to the article and approved the submitted version.
