# Supplementary material for: Dual wave of neutrophil recruitment determines the outcome of C. albicans infection
Source: Front Cell Infect Microbiol. 2023 Jul 10;13:1239593. doi: 10.3389/fcimb.2023.1239593 (PMC10364056; doi:10.3389/fcimb.2023.1239593)
Supplement: Supplementary file 1 [file Image_1.pdf]

**Figure S1**

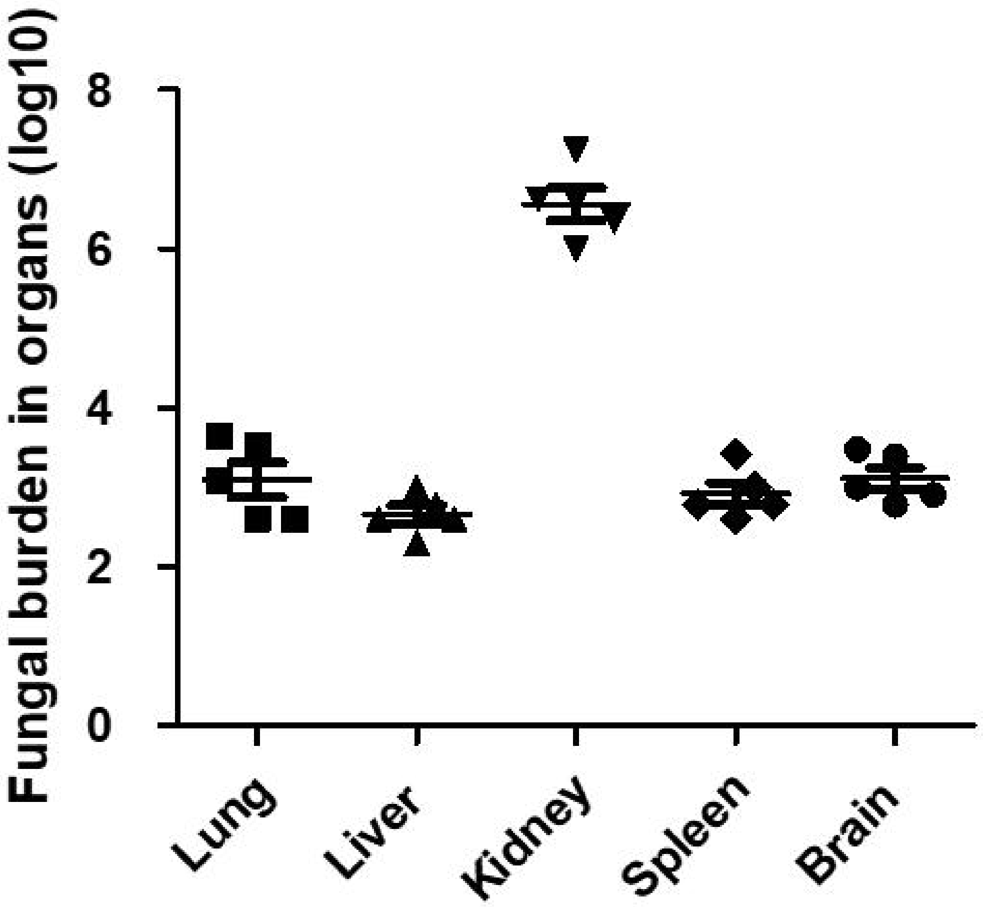


**Figure S1 The number of *C. albicans* in different organs at end time point.** Mice (n=5) were infected with $1x10^5$ *C. albicans* to monitor the survival of mice, The fungal burden in different organs were evaluated when mice showed weight loss of 20 % or found dead or dying.

**Figure S2**

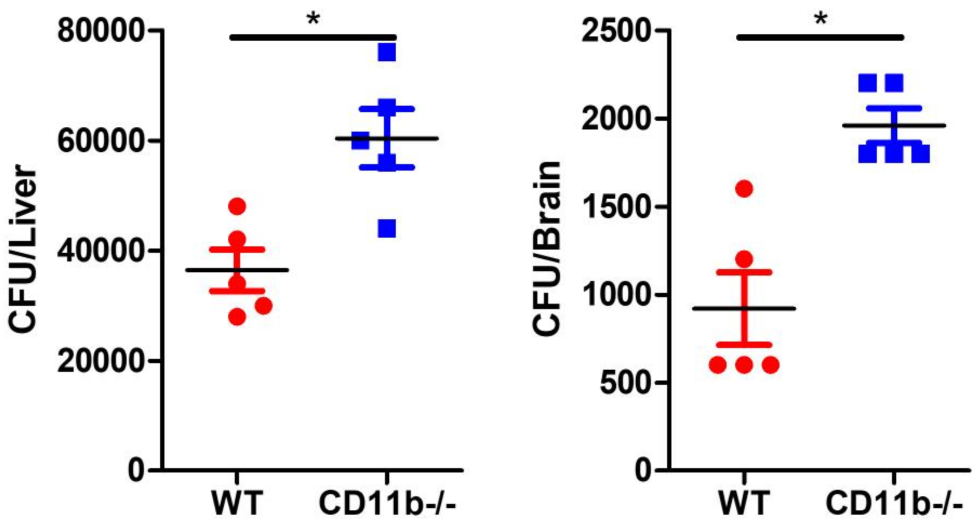


**Figure S2 The fungal burden of liver and brain after i.v injection of $10^6$ *C. albicans*.** Wild type and CD11b$^{-/-}$ mice were euthanized 4 hours after infection (n=5) and the CFU in the liver and the brain was evaluated by serial dilution of the homogenized tissue.

**Figure S3**

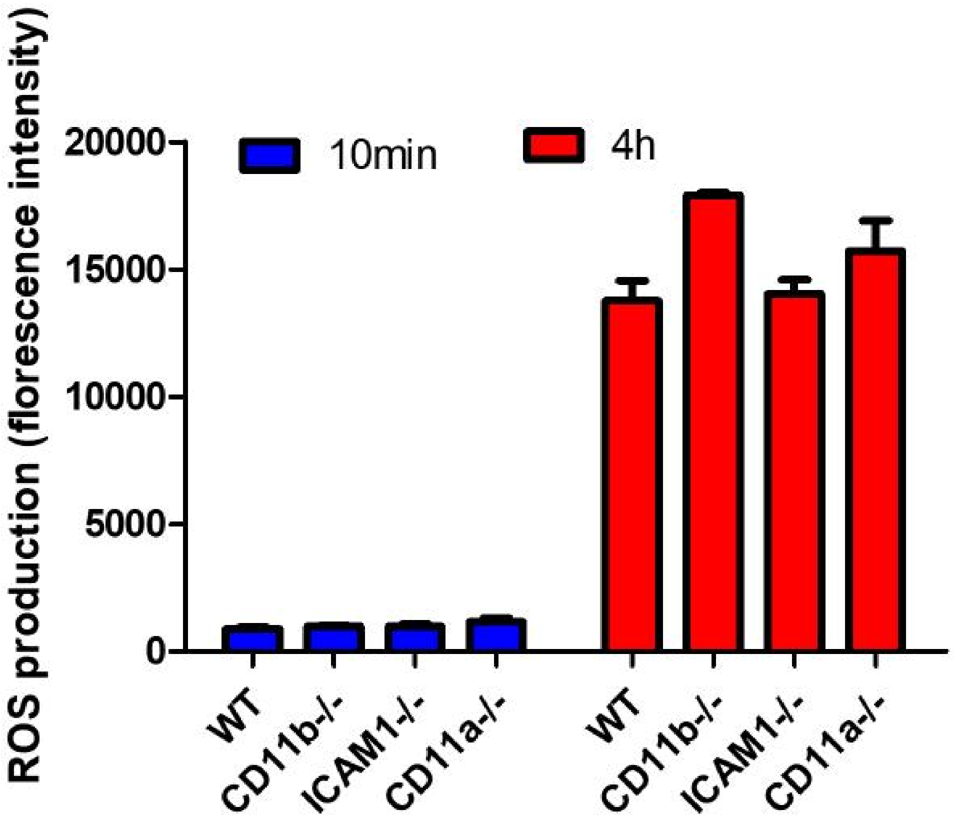


**Figure S3 ROS production as determined by DHR123.** Neutrophils were isolated from wild type or different knockout mice (n=3), and stimulated in vitro in 96 well plates using PMA at final concentration 10 ng/ml for 10 min, the production of ROS was evaluated by DHR123 assay.
